# Accelerated Selective Li+ Transports Assisted by Microcrack‐Free Anionic Network Polymer Membranes for Long Cyclable Lithium Metal Batteries

**DOI:** 10.1002/advs.202308530

**Published:** 2024-02-13

**Authors:** Jingyi Gao, Jiaming Zhou, Xiaodie Chen, Ran Tao, Yao Li, Yu Ru, Chang Li, Eunjong Kim, Xiaoting Ma, Min Wang, Yoonseob Kim, Seungkyu Lee, Dong‐Myeong Shin

**Affiliations:** ^1^ Department of Mechanical Engineering The University of Hong Kong Pokfulam Road Hong Kong 999077 China; ^2^ Department of Chemical and Biological Engineering The Hong Kong University of Science and Technology Kowloon Hong Kong 999077 China; ^3^ Department of Chemistry The University of Hong Kong Pokfulam Road Hong Kong 999077 China

**Keywords:** anionic networks, lithium metal batteries, microcrack‐free, polymer electrolytes

## Abstract

Rechargeable Li metal batteries have the potential to meet the demands of high‐energy density batteries for electric vehicles and grid‐energy storage system applications. Achieving this goal, however, requires resolving not only safety concerns and a shortened battery cycle life arising from a combination of undesirable lithium dendrite and solid‐electrolyte interphase formations. Here, a series of microcrack‐free anionic network polymer membranes formed by a facile one‐step click reaction are reported, displaying a high cation conductivity of 3.1 × 10^−5^ S cm^−1^ at high temperature, a wide electrochemical stability window up to 5 V, a remarkable resistance to dendrite growth, and outstanding non‐flammability. These enhanced properties are attributed to the presence of tethered borate anions in microcrack‐free membranes, which benefits the acceleration of selective Li^+^ cations transport as well as suppression of dendrite growth. Ultimately, the microcrack‐free anionic network polymer membranes render Li metal batteries a safe and long‐cyclable energy storage device at high temperatures with a capacity retention of 92.7% and an average coulombic efficiency of 99.867% at 450 cycles.

## Graphical abstract

Microcrack‐free anionic network polymer membrane (MCf‐ANP) for use as a high‐performing single‐ion conducting electrolyte for long cyclable lithium metal batteries is reported. The presence of tethered anions in MCf‐ANP membrane not only benefits the acceleration of selective Li+ cations transport, but also exceptionally suppresses the dendrite growth over the course of battery cycling, endowing remarkable cycling stability with Li‐metal batteries.

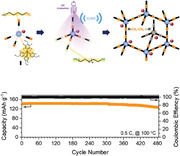

## 1 Introduction

The European Union and the United States have already begun to transition to electric vehicles (EVs) in response to the global climate crisis and the shortage of fossil fuels, with the aim to phase out conventional fuel vehicles by 2030 and 2050, respectively. Shifting to EVs will require more advanced battery technology than current Li‐ion batteries, which only have a capacity of 300 Wh kg^−1^.^[^ ^1^ ^]^ The battery society has put efforts into developing rechargeable lithium metal batteries (LMBs) with more aggressive chemistries to take advantage of Li metal anodes, such as lightweight and high capacity.^[^ ^2^ ^]^ However, the rechargeable LMBs have suffered from undesirable not only lithium dendrite growth but also solid‐electrolyte interphase (SEI) reaction, resulting in safety concerns and a shortened battery cycle life.^[^ ^3^ ^]^ Moreover, many batteries are widely used in consumer applications at room temperature, but there is a growing need for batteries that can operate in extreme thermal environments, such as high temperatures, which are required by various industrial sectors, such as medical devices that need sterilization, subsurface exploration, and thermal reactors.^[^ ^4^ ^]^ Although intensive studies have been conducted to elucidate and sophisticatedly control the dendrite and SEI formations,^[^ ^5^ ^]^ the physics underlying cycle life determination of LMBs remains to be unveiled as the copious parasitic reactions occurring during battery operation are very complex. Several strategies have been introduced to achieve a long battery lifespan, which include but not limited to stabilizing anode,^[^ ^2^, ^6^ ^]^ eliding Li metal (anode‐free),^[^ ^5^, ^7^ ^]^ optimizing anode thickness,^[^ ^8^ ^]^ leveraging novel electrolytes,^[^ ^9^ ^]^ but a stable and long cycle life at high temperature (>400 cycles with >90% capacity retention) has not been accomplished in both coin and pouch cells even at the laboratory level.

The model by J. N. Chazlviel predicted that cell polarization and dendrite growth would be minimized in single‐ion conducting (SIC) electrolytes as lithium can be plated and stripped evenly during the charging/discharging process.^[^ ^10^ ^]^ Recently, there have been numerous reports on experimental demonstrations of SIC electrolytes being resistant to dendrite growth in Li symmetric cells,^[^ ^11^ ^]^ but the LMBs comprising SIC polymer electrolytes have yet to achieve a stable and long lifespan due to persistent issues including internal crack formation and low ionic conductivity at ambient temperature. We reported earlier on a series of interpenetrating porous network polymers that function as viable SIC electrolytes for LMB applications, in which a weakly coordinating borate anion node and polyether bridging unit were processed into a SIC network polymer.^[^ ^12^ ^]^ These polymers exhibited excellent electrochemical stability toward both the Li metal anode and the high‐potential cathode. It also had decent ionic conductivity at ambient temperature, as well as exceptional ion selectivity for conduction and outstanding thermal stability. Importantly, we demonstrated a battery prototype featuring the SIC porous network polymer electrolyte that outperformed a conventional battery in terms of rate capability and resistance to dendrite growth in a symmetric cell. However, a post‐synthetic process to form a membrane yields the inherent microcracks between polymer particles, impeding ionic transport as well as causing the cell polarization, which may be a potential dendrite nucleation site and thus give rise to the capacity fading.

Herein, we report a series of microcrack‐free anionic borate network polymer membranes that serve as a highly efficient electrolyte for safe and long‐cyclable Li‐metal battery applications. We employed organosulfur chemistry to form anionic network polymer (ANP) membranes consisting of perfluorinated tetraphenylborate anions connected through thiol‐ended polyether linkers. The microcrack‐free membranes being tethered borate anions endow the ANP with a selective accelerated Li^+^ cation conduction, facilitating a decent cationic conductivity (σ_Li+_ = 3.1 × 10^−5^ S cm^−1^) at 88 °C, a wide electrochemical window (up to 5 V), an outstanding resistance to dendrite growth, and superior non‐flammability. Remarkably, the battery prototype exhibited an exceptional cyclability performance at high temperatures (>450 cycles with a capacity retention of 92.7%) with average coulombic efficiency of 99.867%. Safety test of the battery prototype demonstrated stable discharging both in the wide temperature range of 30–120 °C and under a negatively pressurized environment, suggesting microcrack‐free anionic borate network polymer membranes render LMBs a stable and sustainable energy storage device.

## 2 Results and Discussion

### 2.1 Click‐Reacted Anionic Borate Network Polymer Membrane

**Figure 1:**
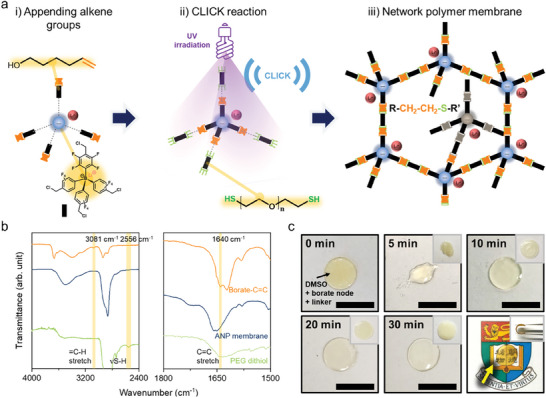
Microcrack‐free anionic network polymer membrane. a) Schematic illustration of a synthetic strategy for anoinc network polymer electrolyte. b) FTIR spectra of borate monomer with alkene groups (orange), anionic network polymer membrane (navy), and PEG dithiol (light green). c) Anionic network polymer gels on the substrate with different UV illumination times. Insets indicate the fully dried membrane. Scale bar is 20 mm. Bottom right displays the representative membrane and inset shows the flexibility of the membrane.

To promote the formation of microcrack‐free network polymer membranes, a series of borate network polymer membranes were directly polymerized on the substrate by the thiol‐ene click chemistry (**Figure** **1**a). The lithium tetrakis(4‐(chloromethyl)−2.3.5.6‐tetrafluorophenyl)borate nodes featuring chloride end groups were first reacted with 5‐hexenol to append the alkene moieties through nucleophilic substitution (Figure 1a‐i). The Fourier‐transform infrared (FTIR) spectra of the modified borate node revealed that the = C─H and C═C stretching peaks clearly appeared at 3081 and 1640 cm^−1^, respectively, whereas the C─Cl bands no longer existed in the frequency range of 600−800 cm^−1^ (Figure S1a, Supporting Information), verifying a successful functionalization.^[^ ^11b^ ^]^ Further corroboration was obtained by the appearance of characteristic peaks in ^1^H and ^13^C nuclear magnetic resonance (NMR) spectra (Figure S1b, Supporting Information). The borate nodes and linkers were dispersed in dimethyl sulfoxide (DMSO) and then the precursor solution was poured into a polydimethylsiloxane mold. Polymerization of the borate nodes with poly(ethylene glycol) (PEG) dithiol under the illumination of ultraviolet (UV) light (Figure 1a‐ii) resulted in the establishment of interpenetrated ANP membranes (Figure 1a‐iii). The direct polymerization was confirmed by the nearly complete disappearance of characteristic alkene peaks of 3081 and 1640 cm^−1^ as well as thiol bands of 2556 cm^−1^ in the FTIR spectrum of the polymer membrane (Figure 1b).^[^ ^13^ ^]^ The UV exposure time was critical in yielding robust membranes, and a sufficient exposure time of >30 min has been shown to generate a transparent and peelable gel from the substrate under 8 watts of power (Figure 1c). The free‐standing gel underwent solvent replacement with methanol and tetrahydrofuran, followed by the baking at 120 °C for 18 h to evaporate solvents under a vacuum fully. The click‐reacted membrane was shown to be transparent, while the membrane fabricated by drop casting was opaque (Figure S2a, Supporting Information), indicating less microcrack formation inside the membrane. The membrane thickness could be controlled by varying the volume of precursor solution (Figure S2b, Supporting Information), and a maximum thickness of ≈356.8 µm with a diameter of 7 mm was obtained. The resulting products of each synthetic and fabrication step were characterized using ^1^H, ^13^C, and ^19^F NMR spectroscopy, FTIR spectrometer, and elemental analysis, which are available in the Supporting information (Figures S3–S6, Supporting Information; Method part).

### 2.2 Ion Conducting Behaviors of Anionic Borate Network Polymer Membrane

**Figure 2:**
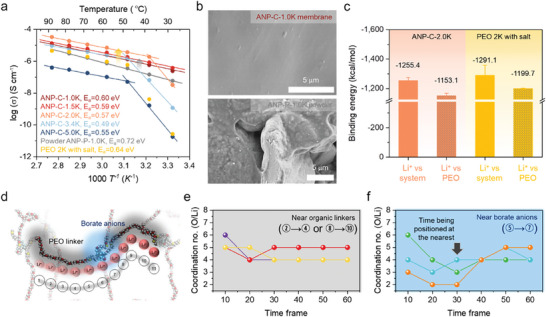
Cation conducting behaviors in the ANP membranes. a) Temperature‐dependent ionic conductivities of the ANP membranes, powder ANP‐P‐1.0K, and PEO 2K with salt. b) The SEM images of ANP‐C‐1.0K membrane and ANP‐P‐1.0K powder. c) Binding energies of Li^+^ cations to ANP‐C‐2.0K and PEO 2K with salt/LiTFSI. d) Schematic of Li^+^ cation transport pathway throughout anionic polymer network. The oxygen coordination number to single Li+ cation in the vicinity of (e) organic linkers and (f) borate anions.

The energetic of Li^+^ conduction was probed via temperature‐dependent impedance measurements, and the Arrhenius plot shown in **Figure** **2**a illustrates the Li^+^ conduction behavior of all membranes. We employed the PEG dithiol linkers with varying molecular weights of 1.0, 1.5, 2.0, 3.4, and 5.0K, and termed the series of ANP membranes with differing linkers as ANP‐C‐nK in which nK represents the molecular weight of the linkers. The ionic conductivity of ANP‐C‐1.0K attained the value of 2.48 × 10^−7^ S cm^−1^ at 28 °C, and this value decreases to 1.81 × 10^−11^ S cm^−1^ with increasing the molecular weights of PEG linkers. We found crossover behavior in the temperature‐dependent ionic conductivity of the ANP membranes with longer linkers, including ANP‐C‐2.0K, ANP‐C‐3.4K, and ANP‐C‐5.0K, which is due in large part to the phase transition from crystalline to amorphous of the polyether linkers upon heating. The phase transition in the membranes, which was confirmed by differential scanning calorimetry (DSC) (Figure S7a, Supporting Information) and temperature variable x‐ray diffractometer (Figure S8, Supporting Information), facilitates the plasticization of the polyether linkers (Figure S9, Supporting Information) and resultingly increases the fractional free volume in the membrane, helping to achieve the improved ionic conductivity of 3.36 × 10^−5^ S cm^−1^ in ANP‐C‐2.0K at 88 °C. We characterized the cation selectivity in the ionic conduction featured by lithium transference number (t_Li+_) in a symmetric Li | ANP‐C‐2.0K | Li cell, and a high t_Li+_ of 0.932 at room temperature reveals that lithium cations are the exclusive mobile species in the membrane (Figure S10, Supporting Information). Lastly, these ANP membranes exhibit moderate activation energies in the range of 0.49–0.60 eV.

### 2.3 Accelerated Li^+^ Cation Transport in Anionic Borate Network Polymer Membrane

Interestingly, the ANP membranes are found to be accelerated Li^+^ cation transports by approximately an order of magnitude compared to powder ANP and PEG, which we likely attribute to two main virtues: 1) hindered microcrack formation and 2) tethered borate anions in the membranes. First, control over the microcrack formation in the membrane gives rise to avoiding the tortuous conduction pathway for Li^+^ cation, hastening ionic conduction. Scanning electron microscopy (SEM) images shown in Figure 2b support this depiction, revealing apparent cracks and gaps for powder ANP, whereas the ANP‐C‐1.0K membrane possesses a consistent and smooth surface. A higher activation energy of E_a_ = 0.72 eV also suggests a restricted series of cation hopping between coordinating sites in powder ANP. These traits imply that the reduced crack formation renders ANP membranes highly ion‐conducting electrolytes.

Moreover, tethered borate anions in the membranes provide not only PEG linker plasticizing but also efficient cation decoupling from the polyether groups in the process of intermediate hopping, engendering improved cationic conduction. Implementation of periodically tethered borate anions into the membrane places the ether chains apart, downshifting the melting point of the PEG linkers (Figure S7b, Supporting Information). The reduced intensity of the crystalline ether band at 1060 and 1145 cm^−1^ in the FTIR spectrum of the membrane corroborates the plasticization of PEG linkers (**Table** **1**; Figure S11, Supporting Information). Furthermore, the amorphous band of ether groups centered at 1094 cm^−1^ is split into Li^+^‐coordinating (low frequency) and non‐coordinating (high frequency) peaks in the polymer membranes, implicating that the complexes of Li^+^ cations and ether groups are constructed after ANP polymerization. Weakly coordinating boron anions in the network polymer likely facilitate the weakening of the binding affinity of such complexes. Density functional theory calculations were employed to determine the binding energy of Li^+^ cations in the ANP membrane, and the results are displayed in Figure 2c. The binding energy of boron anions to Li^+^ cations has been found to be 102.3 kcal mol^−1^, which is comparable to the reported ion pair energy of supramolecular lithium−(C_6_F_5_)_4_B^−^ − Li^+^.^[^ ^14^ ^]^ For comparison, we also evaluated the binding energy of Li^+^ cation in PEG with 0.2 mm lithium bis(trifluoromethanesulfonyl)imide (LiTFSI). The binding energy of polyether groups in the ANP membrane is substantially lower than that of the PEG with salt, smoothing the efficient cation decoupling. Low ion pair energy in the ANP membrane is likely due to the greater tendency of borate anions to pair with Li^+^ compared to TFSI^−^, delocalizing Li^+^ throughout the electrolyte. Thus, a larger amount of dissociated Li^+^ results in more freedom of motion and higher ionic transport. To gain further insight into the environment of Li^+^ cation during its transport throughout the membrane, we investigated the dynamics of Li^+^ cation hopping in the ANPs under the electric field of 0.6 V/Å using molecular dynamics simulations. We found that the Li^+^ cations tend to undergo two different conduction channels, i.e., the borate anion participating ((5) to (7)) and non‐participating paths ((2) to (4) or (8) to (10)) as shown in Figure 2d. Each Li^+^ cation is coordinating with neighboring 4 to 6 oxygen atoms of polyether linker (Figure 2e), and vacancy of Li^+^‐oxygen coordination drives to take place a series of hops along the polymer chains. Interestingly, the oxygen coordination number of Li^+^ has been shown to drop significantly to 2–3 when approaching the borate anions within the distance ranges of 3.36(2)–7.17(7) Å (Figure 2f), weakening ion pairs and allowing the long‐distance hop (Movie S1, Supporting Information). Overall, the linker plasticizing and efficient cation decoupling from the linker together rationalizes the accelerated Li^+^ cations transport observed in the boron anion tethered ANP.

**Table 1:** Ether vibration bands of ANP‐C‐nK membranes and bare PEO linker.

Vibration assignment		PEO linker^[^ ^26^ ^]^	ANP‐C −5.0K	ANP‐C−3.4K	ANP‐C−2.0K	ANP‐C−1.5K	ANP‐C−1.0K
ν_s_(C‐O‐C)	Peak center (cm^‒1^)	1145	1144.6	1143.5	1142.2	1143.0	1142.8
	FWHM (cm^‒1^)		20.0	22.2	25.4	25.1	25.1
ν_s_(C‐O‐C)	Peak center (cm^‒1^)	1093	1102.9	1103.3	1105.9	1114.9	1117.0
	Area		16.11	15.75	14.28	7.94	6.06
ν_s_(C‐O‐C) |Li	Peak center (cm^‒1^)		1078.4	1080.8	1083.2	1087.1	1088.9
	Area		3.71	7.51	12.78	15.03	16.00
ν_s_(C‐O‐C)	Peak center (cm^‒1^)	1060	1059.9	1060.1	1060.9	1066.4	1067.5
	FWHM (cm^‒1^)		10.2	9.3	11.7	14.1	13.8

ν_s_ = symmetrical stretch, Crystalline bands: 1145 and 1060 cm^−1^, Amorphous band: 1094 cm^−1^

### 2.4 Electrochemical, Thermophysical, and Mechanical Stability of ANP Membrane

**Figure 3:**
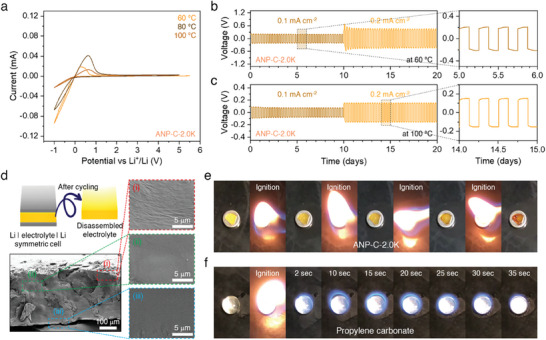
Electrochemical and thermophysical stability of ANP‐C‐2.0K membrane. a) Oxidation stabilities at different temperatures of 60, 80, and 100 °C. Potential profiles over the course of lithium plating/stripping in a symmetric Li|electrolyte|Li cell with current densities of 0.1 and 0.2 mA cm^−2^ at b) 60 and c) 100 °C. d) Cross‐sectional SEM images of membrane after 160 cycles of galvanostatic polarizations. Combustion test of e) ANP‐C‐2.0K and f) propylene carbonate.

As the ANP‐C‐2.0K membrane showed a standout selective cation‐conducting performance, subsequent measurements denoted below using the ANP membrane were all performed with the ANP‐C‐2.0K membrane. The oxidative stability of the ANP membrane was assessed using cyclic voltammetry on stainless steel electrodes, in which the measurements were conducted in voltages ranging from −1.0 to 5.5 V (vs Li/Li^+^) with a scan rate of 0.3 mV s^−1^ at varying temperatures of 60, 80, and 100 °C. The ANP membrane exhibited a low oxidative current and exceptional stability up to 5.0 V versus Li/Li^+^ even at elevated temperatures (**Figure** **3**a). This enhanced stability may arise as a result of the restricted decomposition of stationary anions in tandem with reduced defects of the ANP membrane, which together minimize the oxidation decomposition at the electrolyte–electrode interface and thus enable to surpass the values reported in the literature for anionic network polymers (4.2 or 4.5 V vs Li/Li^+^).^[^ ^12^ ^]^ The wide electrochemical window stability at high temperatures suggests the compatibility of the ANP membrane for potential application into Li‐metal batteries operating at extreme thermal conditions. The dynamic stability of the Li | ANP membrane interface at temperatures of 60 and 100 °C was further examined using galvanostatic lithium plating/stripping electrochemical cycling measurements (Figure 3b,c). A practical cycling process was emulated by implementing a 3 h lithium plating followed by a 3 h lithium stripping with current densities of 0.1 and 0.2 mA cm^−2^. The symmetric cells demonstrated a notably stable voltage polarization during the successful plating and stripping of lithium for 20 days. The resistance to dendrite growth has been quantified with the concept of total charge passed, C_d,_^[^ ^5f^ ^]^ and the values of C_d_ were 86.4 and 172.8 C cm^−2^ for the current densities of 0.1 and 0.2 mA cm^−2^, respectively. The ANP membrane showed a stable charge transport over the course of cycles for 40 days at different current densities and temperatures, achieving C_d_ of 518.4 C cm^−2^. The ANP membrane is shown to outperform conventional PEG electrolytes (low *C_d_* values of ≈5 to 18.2 C cm^−2^ at various current densities in the range of 0.17–1.0 mA cm^−2 [^ ^5^, ^15^ ^]^) as well as the drop‐casted ANP membranes (*C_d_* values of 51.8 to 216 C cm^−2^ in the range of 0.01–0.25 mA cm^−2[^ ^12^, ^16^ ^]^). It is noteworthy that those values of ANP membrane can be further increased, given that the values were not obtained at the time of cell failure as the symmetric cell showed no cell failure for 40 days in total. The SEM images visualize the resistance to dendrite growth in the membrane that underwent 160 cycles of galvanostatic lithium plating/stripping measurements (Figure 3d) and revealed the absence of noticeable damage throughout the electrolyte membrane, supporting the successful suppression of lithium dendrite growth obtained by electrochemical characterization.

We also measured the thermal stability of the ANP membrane using thermogravimetric analysis (TGA) (Figure S12, Supporting Information). The membrane was stable up to ≈300 °C, and began to decompose in the temperature range of ≈300 to 400 °C associated with the thermal decomposition of polyether^[^ ^17^ ^]^ and anionic tetrakis(pentafluorophenyl)borate.^[^ ^18^ ^]^ The flame tests depicted that the ANP membrane was not ignited even with multiple applications of an open flame (Figure 3e; Movie S2, Supporting Information) and resultingly simply turned into coal ash, in sharp contrast with the highly flammable carbonate liquid electrolyte (Figure 3f), indicating that the ANP membrane exhibits excellent fire retardancy before thermal decomposition. The flame retardancy of the ANP membrane is attributed to the presence of abundant fluorines, which are difficult to associate with oxygen, in borate anions, and indeed fluorination has previously been employed to reduce the flammability of various electrolytes.^[^ ^5^, ^19^ ^]^ Meanwhile, mechanical tests suggested that the membrane behaves as a soft elastic solid with Young's modulus of 20.42 MPa (Figure S13, Supporting Information) and shear modulus of 31.9 ± 0.5 kPa (Figure S14, Supporting Information), benefiting the relaxation of significant stresses from electrode materials. The membrane becomes further softer with increasing temperature from 25 to 60 °C as demonstrated by the decreased shear modulus down to 7.52 ± 0.44 kPa, which is predicted to facilitate better interfacial adhesion between electrodes and electrolytes. The flexibility of membrane electrolyte has been demonstrated by the fact that the membrane exhibited nearly consistent resistance irrespective of its bending or flatting status after several cycles (Figure S15, Supporting Information), and the variation in resistance during the bending was approximately 23.7 ± 10.5%. Ultimately, the remarkable thermophysical and mechanical stabilities demonstrate the applicability of the ANP membrane in Li‐metal batteries that operate safely at high temperatures.

### 2.5 Battery Performance at High Temperatures

**Figure 4:**
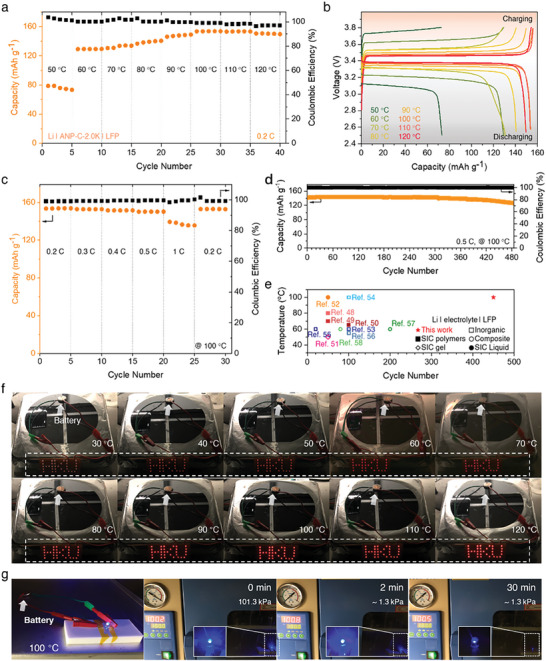
Performance of the full cell with structure of Li|ANP‐C‐2.0K|LFP. a) Capacity performance and b) corresponding charging and discharging profiles at 0.2 C with various temperatures. c) Energy capacity at 100 °C over many charge/discharge cycles at the various rates. d) Cycle performance at 0.5 C and 100 °C. e) Opertating temperature of battery as a function of cycle number (data from Figure 4d) compared with literature data for state‐of‐the‐art electrolytes for Li|LFP batteries: SIC polymers,^[^ ^20^ ^]^ SIC gels,^[^ ^21^ ^]^ SIC liquid,^[^ ^22^ ^]^ inorganics,^[^ ^23^ ^]^ and composites.^[^ ^24^ ^]^ f) Thermal abuse test upon continuous heating. g) Performance at reduced pressure and high temperature of 100 °C.

To verify the compatibility of the ANP membrane as a high‐performing electrolyte in the Li‐metal battery cell, Li|ANP membrane|LiFePO_4_ (LFP) batteries (1C = 170 mAh g^−1^) were assembled and assessed. The battery cell exhibited an excellent specific capacity of 129.3 mAh g^−1^ at 60 °C at a rate of 0.2C (**Figure** **4**a,b), and this value increased with increasing the temperature, achieving 150.3 mAh g^−1^ at 120 °C. Furthermore, the battery cell delivered satisfactory capacities of 76.3 mAh g^−1^ with a well‐defined potential plateau at 50 °C. The overpotential curves were obtained by subtracting the discharge from the charge profile (Figure S16, Supporting Information), and the internal resistance drop and ohmic overpotential are determined as overpotentials at the state of charge (SoC) of 100% and the plateau (SoC of 80‐20%), respectively. Both overpotentials increased linearly with the resistivity of the ANP membrane, indicating that the reduced potential and capacities are mainly attributed to the grown resistivity of electrolytes in the battery cell at different temperatures. The specific capacities of the battery at varying rates of 0.2, 0.3, 0.4, 0.5, and 1.0 C at 100 °C were determined to be 155.2, 153.9, 152.5, 150.8, and 138.1 mAh g^−1^, respectively (Figure 4c; Figure S17, Supporting Information). Remarkably, the capacity was recovered to 153.0 mAh g^−1^ after 5 cycles at each C rate, indicating decent rate capability. Furthermore, the rate performances of the battery cell were carried out at different temperatures of 60, 90, and 110 °C as depicted in Figures S18–S20 (Supporting Information), demonstrating reversible charge–discharge capacity after cycling at different rates under varying high temperatures. After 120 cycles in total at varying rates and temperatures, we disassembled the battery cells to inspect the degradation and decomposition of the ANP membrane. Cross‐sectional SEM images testify to the exceptional resistance to dendrite growth and the viable adhesion of the ANP membrane to both electrodes (Figure S21, Supporting Information), demonstrating favorable compatibility and safety of ANP membrane for high‐temperature battery applications. The long‐term cycling performance was assessed at a discharge rate of 0.5C and a temperature of 100 °C. Figure 4d displays that the battery cell exhibited an average coulombic efficiency (CE) of 99.869% during 485 cycles. The initial charge‐specific capacity reached 141.7 mAh g^−1^, and the value slightly decreased down to 126.6 mAh g^−1^ after 485 cycles, with a capacity retention rate of 89.3%. It is worth noting that more than 92.7% of capacity is impressively retained throughout 450 cycles, demonstrating outstanding long‐term stability of battery cell at high temperature. To our knowledge, this cyclability at high temperature is higher than any value reported to date for Li metal | LFP batteries featured with single‐ion conducting polymers,^[^ ^20^ ^]^ single‐ion conducting gel polymers,^[^ ^21^ ^]^ single‐ion conducting liquid,^[^ ^22^ ^]^ inorganic,^[^ ^23^ ^]^ and composite^[^ ^24^ ^]^ electrolytes (Figure 4e and **Table** **2**). The retarded crack formation in tandem with anchored borate anions in the ANP membranes impedes Li dendrite growth in the electrolyte and engenders homogeneous solid electrolyte interphase on the Li anode, which together remarkably prolongs the battery life span.

**Table 2:** Electrolyte type, discharge rate, operating temperature, initial capacity, cycles, and capacity retention at the last cycle of Li|electrolyte|LFP batteries reported in the literature to date.

Author	Electrolytes	Discharge rate [C]	Temp. [ °C]	Initial capacity [mAh g^‒1^]	Cycles	Capacity retention [%]
This work	Polymer (Single ion)	0.5	100	141	450	92.7
Ref. [19a]	Polymer (Single ion)	0.1	80	128.8	50	99.6
Ref. [19b]	Polymer (Single ion)	0.2	70	140	50	86.0
Ref. [19c]	Polymer (Single ion)	0.2	65	135	100	81.5
Ref. [20]	Gel polymer (Single ion)	0.2	50	156.5	50	88.0
Ref. [21]	Liquid (Single ion)	0.2	100	165	50	89
Ref. ^[^ ^22^ ^a]^	Inorganic	0.1	60	146.2	100	99.4
Ref. [22b]	Inorganic	1	100	126	100	97.6
Ref. [22c]	Inorganic	0.05	60	152	20	72.3
Ref. [22d]	Inorganic	0.1	55	156.5	100	88
Ref. [23a]	Composite	0.1	60	141.5	200	73.2
Ref. [23b]	Composite	0.5	50	146	50	95.6

To verify the safety of Li|ANP membrane|LFP batteries during thermal abuse, the battery temperature is raised up to 120 °C with 10 °C intervals, and we hold the battery at each temperature for 5 min (Figure 4f). The red light‐emitting diodes (LEDs) with character patterns of “HKU” were continuously and stably lit up in the temperature range of 30–120 °C, and no noticeable swelling or deformation was found in the coin cell during the test. Even after 525 cycles at 100 °C at a rate of 0.5C, the battery is still stably operational in the temperature range of 30–120 °C (Figure S22, Supporting Information). Furthermore, the coin cell was exposed to diminished pressure and boosted temperature to mimic the harsh operating conditions (Figure 4g). The coin cell demonstrated stable operation for more than 30 min under an absolute pressure of 1.3 kPa at 100 °C, thus affirming its potential for operation in a high temperature and a high altitude. Undoubtedly, the microcrack‐free ANP membranes will empower impressive cyclability and safety at high temperatures in practical Li metal batteries.

## 3 Conclusion

In conclusion, we have reported a microcrack‐free anionic network polymer membranes, that exhibited a decent cationic conductivity at high temperatures, a wide working window, outstanding resistance to dendrite growth, and superior non‐flammability. Through a combination of hindering microcrack formation and implementing immobile borate anions in network polymers, our findings demonstrate that microcrack‐free ANP membranes can greatly improve the cyclability and safety of Li metal batteries at high temperature, surpassing the Li|electrolyte|LFP batteries with state‐of‐the‐art electrolytes: Furthermore, the presence of tethered borate anions in microcrack‐free ANP membrane not only benefits the acceleration of selective Li^+^ cations transport, but also exceptionally suppresses the dendrite growth over the course of battery cycling. This strategy of enhancing cyclability and safety of Li metal batteries through incorporation of microcrack‐free anionic network polymer membranes is broadly applicable to other high energy density batteries, potentially paving the way for future advancements in the design of anionic electrolytes for the next‐generation lithium batteries.

## 4 Experimental Section

### Microcrack‐Free ANP‐C‐nK Membrane Fabrication

The thio‐enene reaction was employed to form anionic PAF membranes. The alkene‐ended borate anion nodes and thiol‐ended polyether linkers were dissolved in dimethyl sulfoxide with the molecular ratio of 1:2. The weight percentage of mixture solution was ≈50%. 2,2‐Dimethoxy‐2‐phenylacetophenone (DMPA) (1% wt) was added into the mixture solution as a radical initiator, and then the solution was transferred into vacuum vessel to totally remove bubbles inside. The functionalized borate anions and ether linkers will be reacted on the polydimethylsiloxane substrates under ultraviolet light illumination to yield microcrack‐free anionic PAFs. A yellowish membranes formed were subjected to a series of solvent washes. Solvent was exchanged by decanting after periods of at least 6 h: 3 × 6 mL of methanol at 60 °C; and 3 × 6 mL of tetrahydrofuran at 60 °C. After the last solvent wash was removed, the membranes were dried at 60 °C to remove most of the solvent, and further dried at 120 °C under reduced pressure for at least 18 h to fully remove trace of solvent. A thickness of ≈120 µm was chosen for all characterizations.

### Electrolyte Characterizations

The multipotentiostat (Amiral Squidstat Plus) housed in an Ar‐filled glove box was used to investigate the ionic conductivity of samples. The samples were sandwitched between stainless‐steel electrodes to carry out impedance spectroscopy measurement as a function of frequency in the range of 1 Hz–1 MHz at the variable temperatures. The temperature‐dependent ionic conductivities were analyzed to determine the activation energy of the samples using the Arrhenius and Nernst‐Einstein equations. The current response was recorded as a function of time upon applying a 100‐mV dc voltage to Li|electrolyte|Li symmetric cell, and the transference number was calculated using the relationship proposed in ref. [25] Cyclic voltammetries of Li|electrolyte|stainless‐steel cells were carried out between −0.5 and 5.2 V (versus Li^+^/Li) with the scan rate of 0.3 mV s^−1^. The Galvanostatic cyclings were performed on Li|electrolyte|Li cells at selected current densities of 0.1 and 0.2 mA cm^−2^ using a 3 h lithium plating followed by a 3 h lithium stripping.

### Battery Test

To fabricate the composite cathode, a slurry was prepared by mixing LiFePO_4_ (active material, 60 wt%), electrolyte (20 wt.%), conductive carbon black (10 wt.%), and poly(vinylidene fluoride) (10 wt.%) in N‐methylpyrrolidone. The homogeneous cathode slurry was then cast on an Al foil using a doctor blade, and then was dried under a vacuum at 80 °C for 12 h. The electrolyte membrane was sandwiched between the composite cathode and a Li foil anode. The typical mass loading of the active cathode material was ≈1.0 mg cm^−2^. The rate capability and cycle life of battery cells were measured in the voltage range of 2.5–3.8 V at different temperature. The C rates in all of the electrochemical measurements were defined on the basis of 1 C = 170 mA g^−1^.

### Molecular Dynamics Simulation

All calculations were carried out in Materials Studio 2020. The initial simulation structures with 8 Li^+^ atoms and 8 anionic network polymers were built by Materials visualizer. The borate anions and Li^+^ were set as one negative charge and one positive charge, respectively. The simulations were performed in NPT ensemble, and a time step of 1fs was used. Temperature was controlled by application of Andersen thermostat. The systems were heated to 343 K for at least 2 ns to simulate the experimental preparation temperature. The annealing procedures were employed to achieve ion equilibrium in the temperature range from 298 to 600 K with 50 K interval for 50 ps, and were repeated five times. Electrostatic interaction and Van der waals interaction adopted Ewald summation and Atom‐based summation, respectively, with a cut‐off distance of 9.5 Å. Simulations of Li transport under applied the electric field of 0.6 V/Å along the z axis, and sapshots of the trajectory were recorded every 1fs. The Binding energy between the Li^+^ cations and the network systems was calculated using the Equation (1):

(1) $$E_{\mathrm{interaction}}=E_{\mathrm{total}}-\left(E_{\mathrm{Li}}+E_{\mathrm{network}}\right)$$

where the E_total_, E_Li_, and E_network_ are the whole systems energy, the Li^+^ energy, and the network energy in the periodic structure, respectively. Full simulation details are described in Supporting Information.

## Conflict of Interest

The authors declare the following financial interests/personal relationships which may be considered as potential competing interests: the University of Hong Kong has applied for a patent (US application no. 63/598,788) on some of the technology discussed herein, on which some of the authors of this paper are listed as co‐inventors.

## Supporting information

Supporting Information

Supplemental Movie 1

Supplemental Movie 2

## Data Availability

The data that support the findings of this study are available from the corresponding author upon reasonable request.
