# Supplementary material for: Accelerated Selective Li+ Transports Assisted by Microcrack‐Free Anionic Network Polymer Membranes for Long Cyclable Lithium Metal Batteries
Source: Adv Sci (Weinh). 2024 Feb 13;11(17):2308530. doi: 10.1002/advs.202308530 (PMC11077682; doi:10.1002/advs.202308530)
Supplement: Supplementary file 1 — Supporting Information [file ADVS-11-2308530-s001.pdf]

ADVANCED SCIENCE
Open Access

Supporting Information

for *Adv. Sci.*, DOI 10.1002/advs.202308530

Accelerated Selective Li+ Transports Assisted by Microcrack-Free Anionic Network Polymer Membranes for Long Cyclable Lithium Metal Batteries

*Jingyi Gao, Jiaming Zhou, Xiaodie Chen, Ran Tao, Yao Li, Yu Ru, Chang Li, Eunjong Kim, Xiaoting Ma, Min Wang, Yoonseob Kim, Seungkyu Lee and Dong-Myeong Shin**

Supporting Information

Accelerated selective Li+ transports assisted by microcrack-free anionic network polymer membranes for long cyclable lithium metal batteries

*Jingyi Gao, Jiaming Zhou, Xiaodie Chen, Ran Tao, Yao Li, Yu Ru, Chang Li, Eunjong Kim, Xiaoting Ma, Min Wang, Yoonseob Kim, Seungkyu Lee, Dong-Myeong Shin**

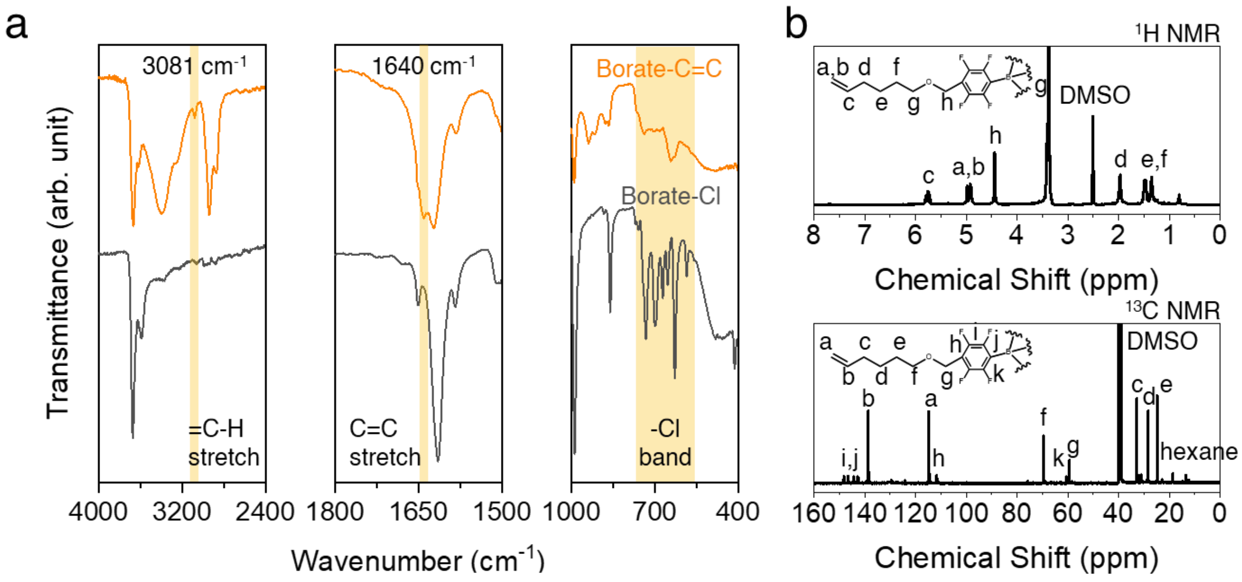

Supplementary Figure 1 | a) FITR spectra of borate monomer and borate monomer with alkene moieties. b) ^{1}H NMR and ^{13}C NMR spectra of borate monomer with alkene moieties in DMSO-d_6.

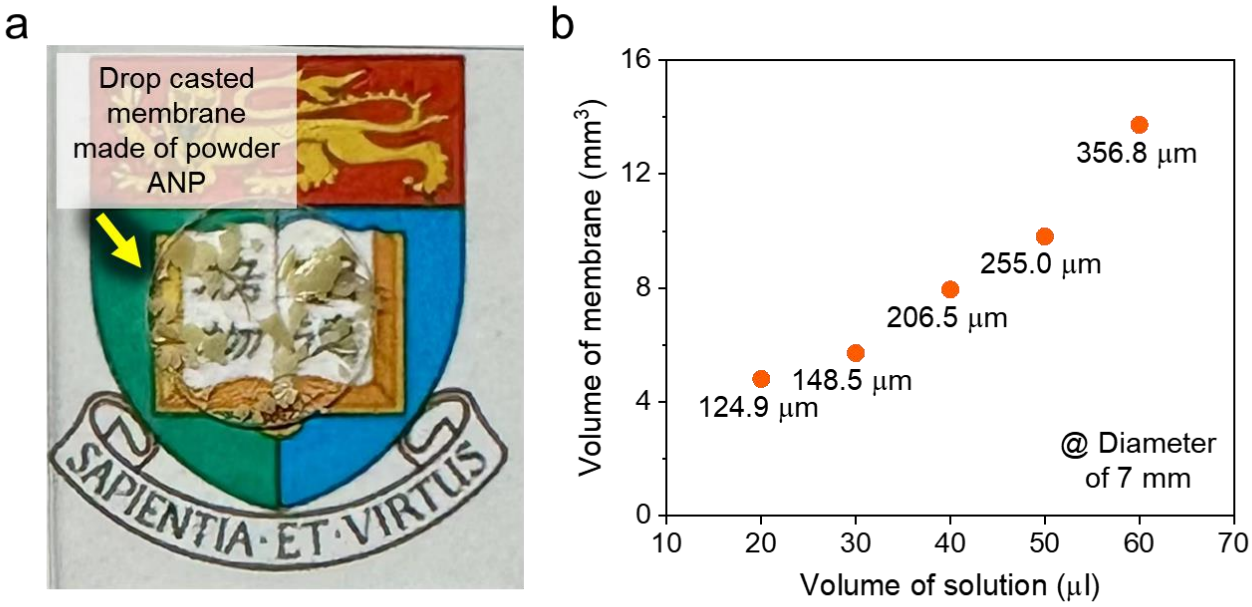

Supplementary Figure 2 | a) Optical image of drop-casted membrane made of powder ANP. b) Volume of membrane as a function of volume of precursor solution. Values indicate the membrane thickness when the diameter is 7 mm as an example.

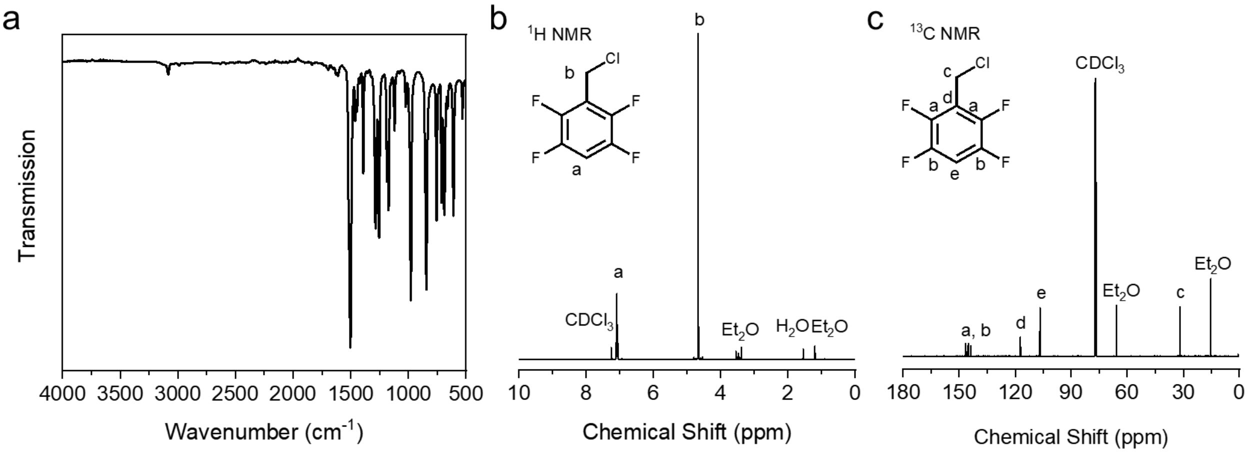

Supplementary Figure 3 | Characterizations of 2,3,5,6-tetrafluorobenzyl chloride. a) FT-IR spectrum. b) ^{1}H NMR spectra in $CDCl_3$. c) ^{13}C NMR spectra in $CDCl_3$.

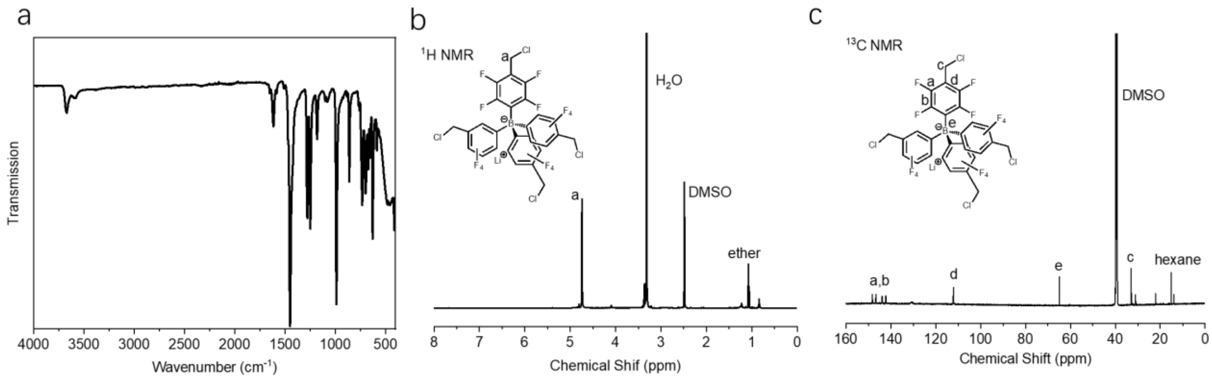

Supplementary Figure 4 | Characterizations of lithium tetrakis(4-(chloromethyl)-2,3,5,6 tetrafluorophenyl)borate. a) FT-IR spectrum. b) ^{1}H NMR spectra in DMSO-d_6. c) ^{13}C NMR spectra in DMSO-d_6.

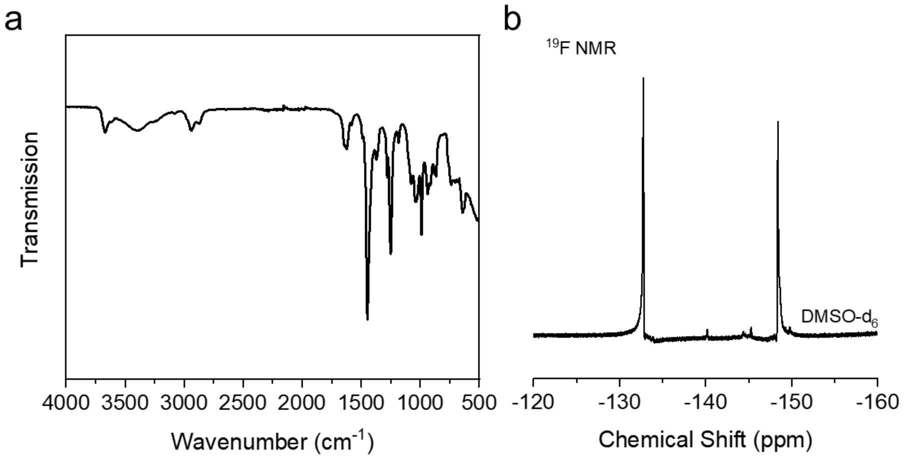

Supplementary Figure 5 | Characterizations of lithium tetrakis(4-(chloromethyl)-2,3,5,6-tetrafluorophenyl)borate with alkene (Borate node-C=C monomer). a) FT-IR spectrum. b) ^{19}F NMR spectra in DMSO-d_6.

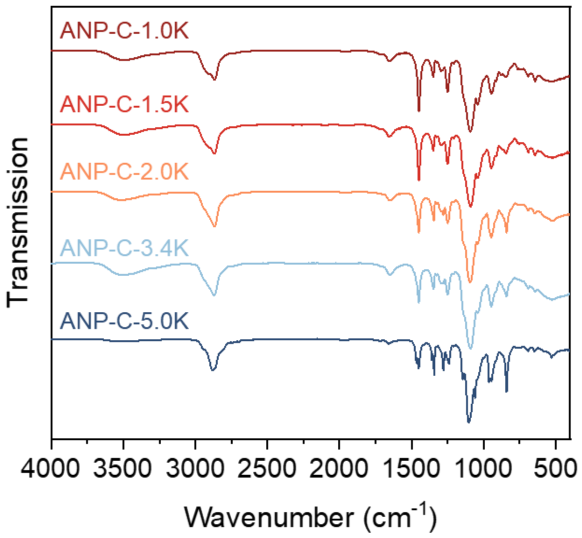

Supplementary Figure 6 | FT-IR spectrum of ANP-C-nK membranes.

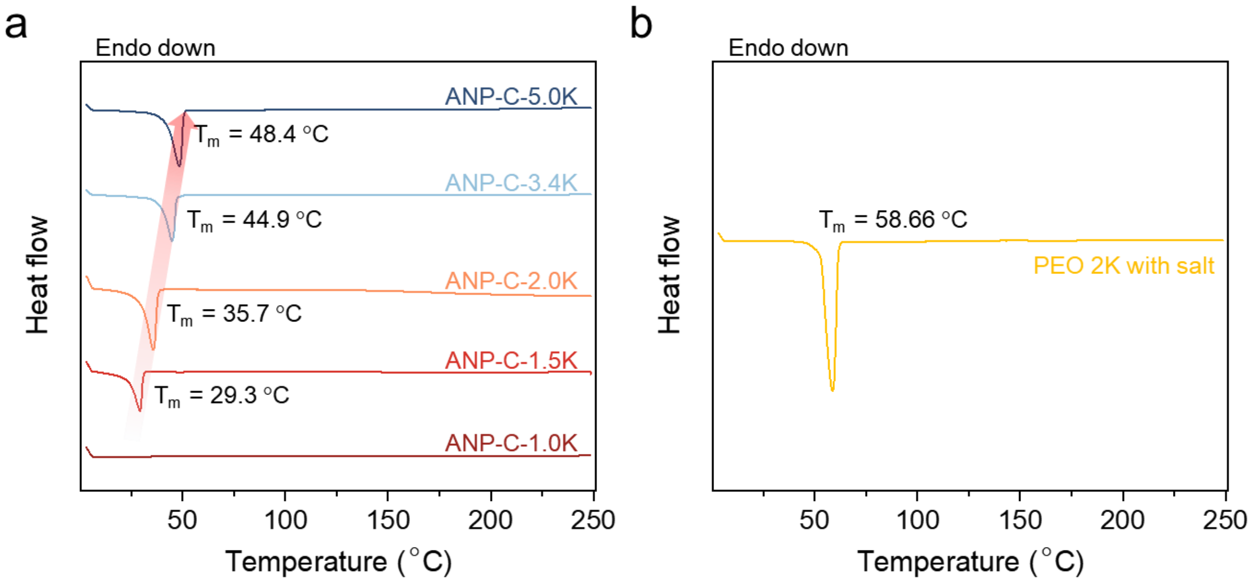

Supplementary Figure 7 | DSC curves of a) ANP membranes and b) PEO 2K with salt.

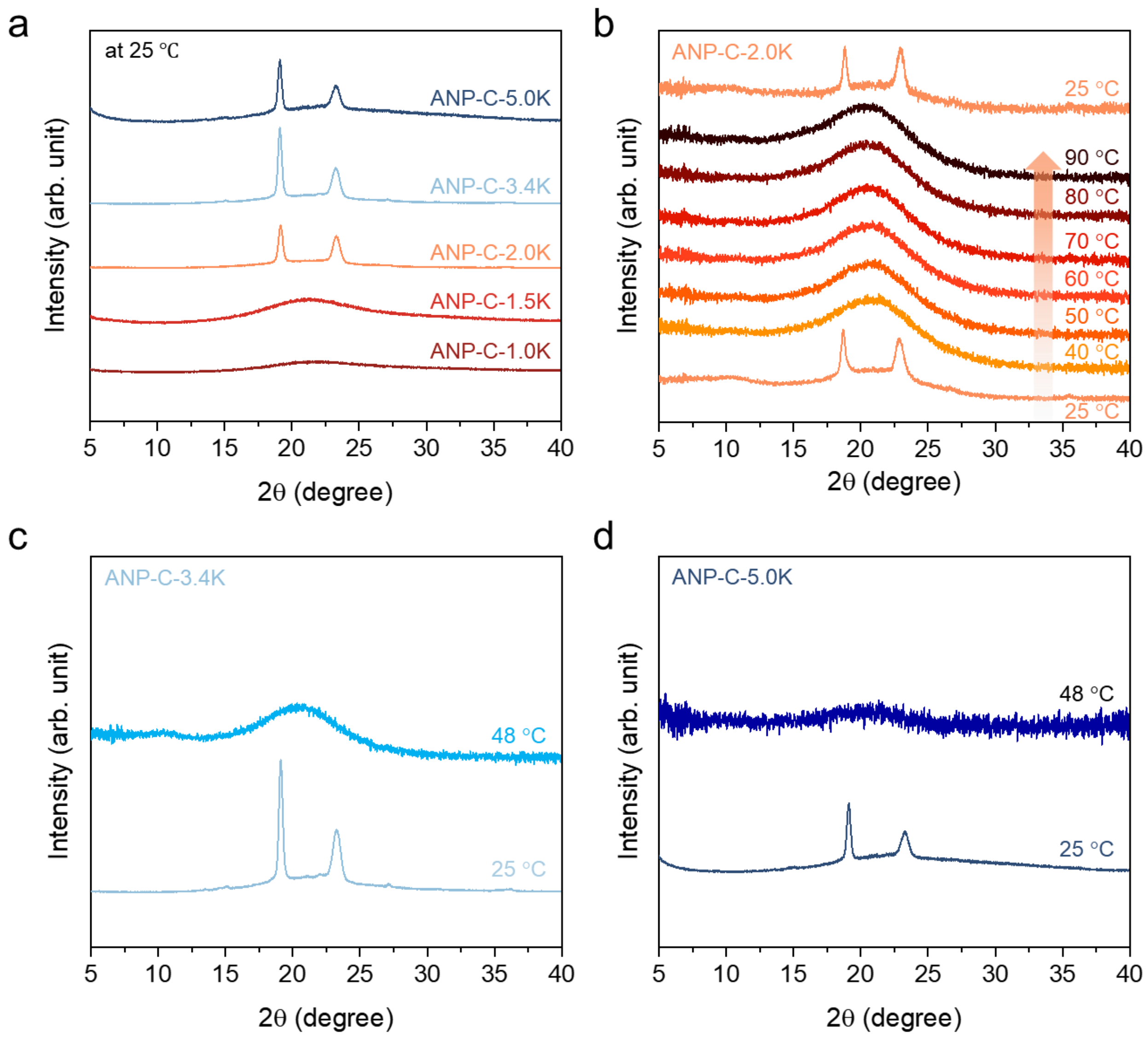

Supplementary Figure 8 | a) XRD patterns of ANP membranes at 25 °C. b) XRD patterns of ANP-C-2.0K at evalutated temperatures. XRD patterns of c) ANP-C-3.4K and d) ANP-C-5.0K at 25 and 48 °C, which correspond to below and above melting temperature, respectively.

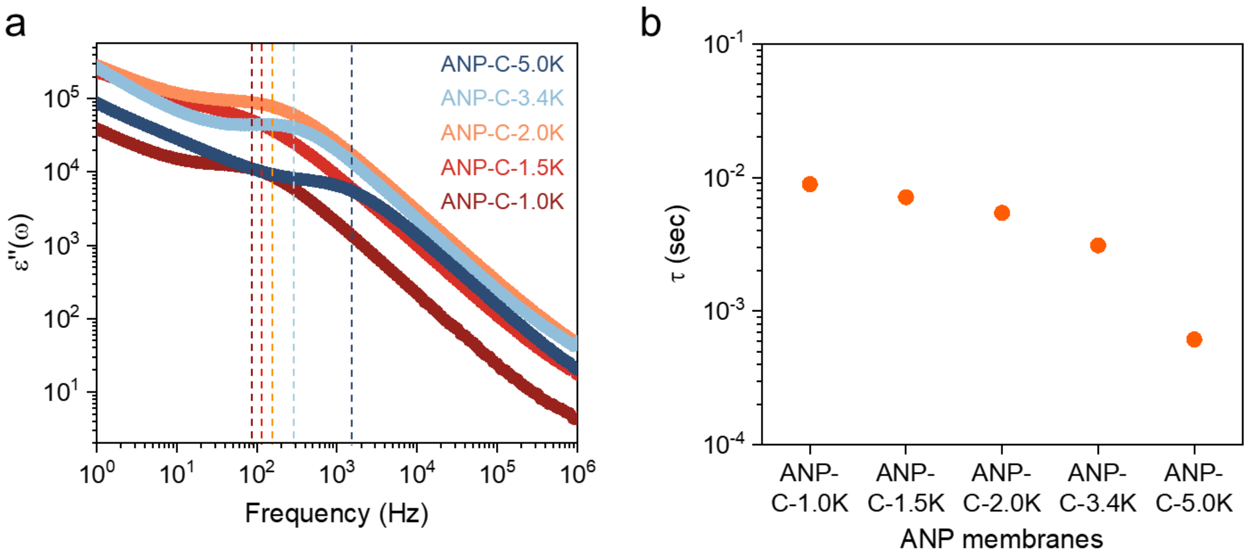

Supplementary Figure 9 | a) Dielectric loss spectra and b) corresponding relaxation times of ANP-C-nK membranes at 25 °C.

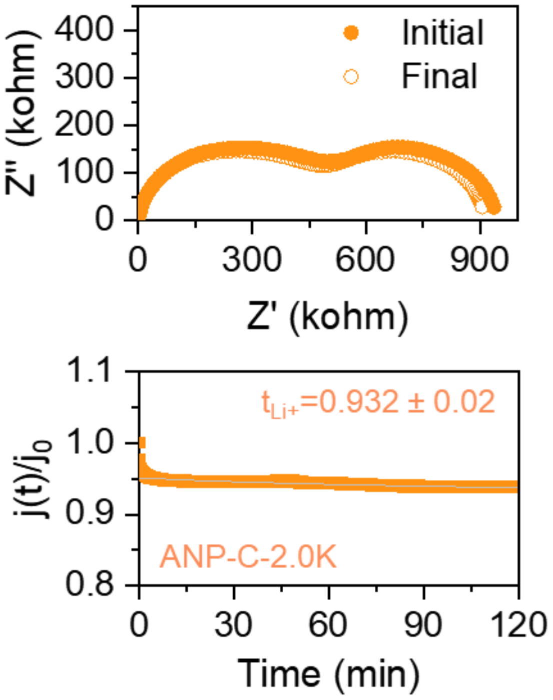

Supplementary Figure 10 | The selectivity in cation conduction of ANP-C-2.0K. The impedance spectra (top) before and after polarization (filled and open symbols, respectively) and current decay curves (bottom).

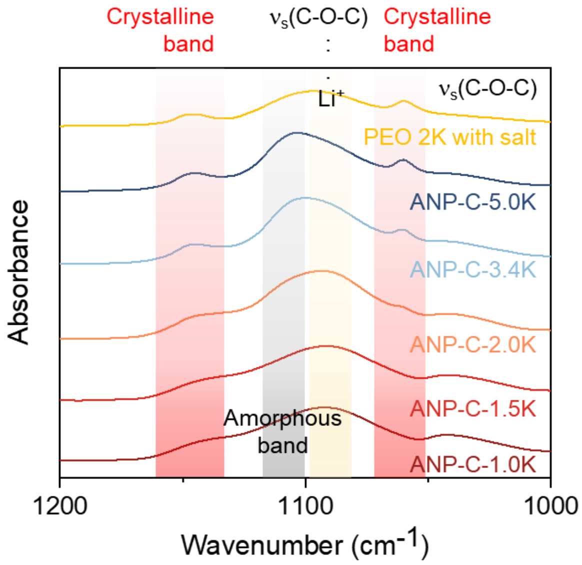

Supplementary Figure 11 | Absorbance spectra of ANP membranes and PEO 2K with salt in the wavenumber range of 1200 to 1000 cm^{-1}, which correspond to ether band.

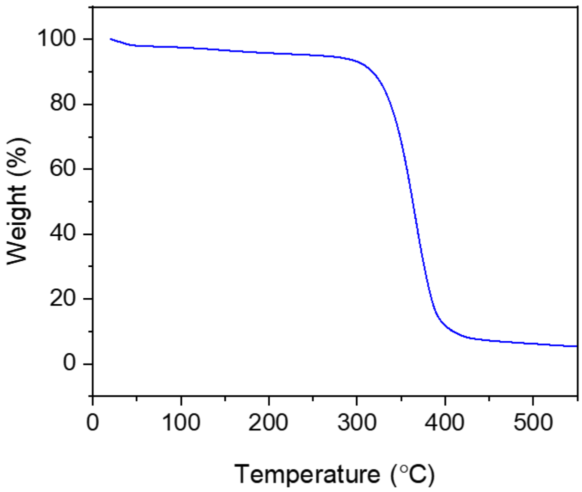

Supplementary Figure 12 | TGA results of ANP-C-2.0K membrane.

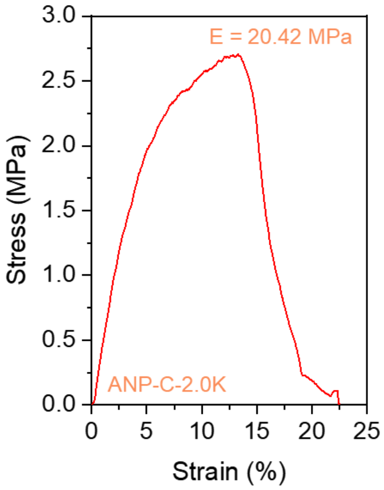

Supplementary Figure 13 | Stress–strain curves of ANP-C-2.0K.

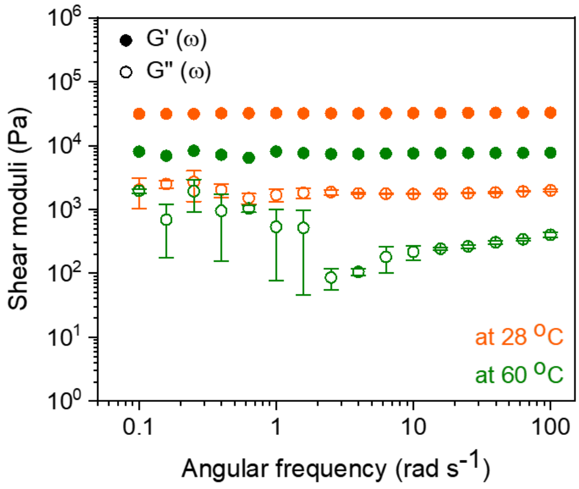

Supplementary Figure 14 | Frequency dependency of the storage (G′) and loss (G″) moduli for ANP-C-2.0K membrane at 28 °C and 60 °C.

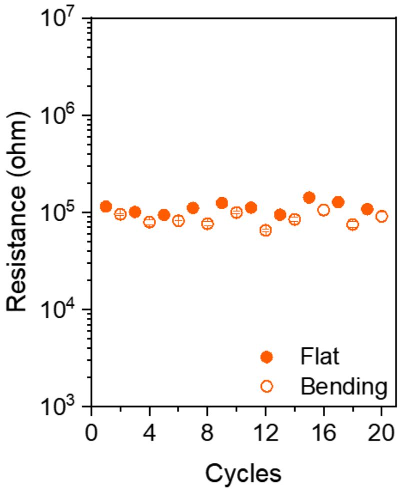

Supplementary Figure 15 | Resistance of ANP-C-2.0K after bending several times.

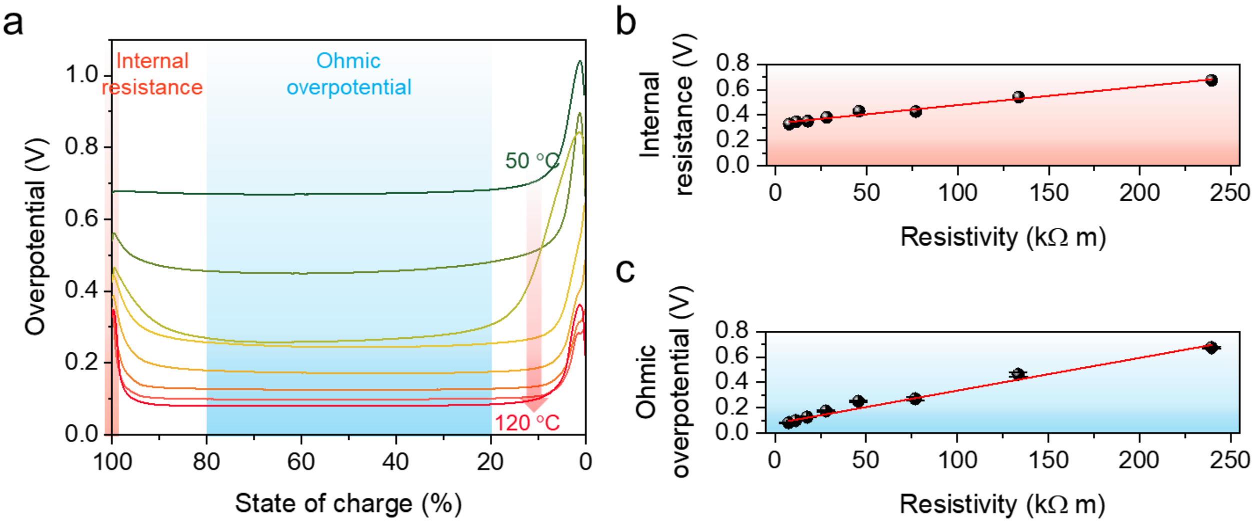

Supplementary Figure 16 | a) Overpotential of battery as a function of state of charge (SoC) at different temperatures. b) Internal resistance as a function of measured resistivity for ANP-C-2.0K membrane. Internal resistance was defined as the overpotential at 100 % SoC. c) Ohmic overpotential as a function of measured resistivity for ANP-C-2.0K membrane. Ohmic overpotential was defined as the overpotential in the SoC range of 20-80 % SoC.

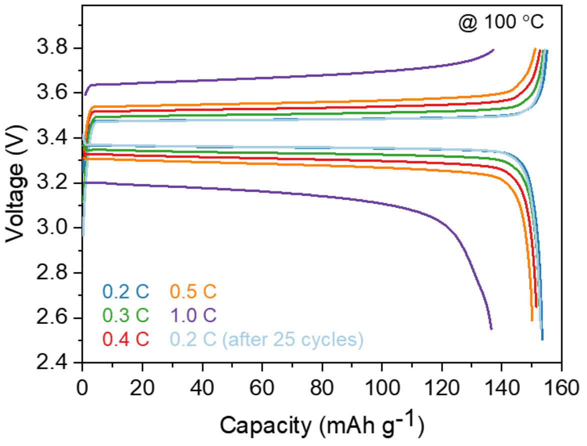

Supplementary Figure 17 | Charging and discharging profiles of Li|ANP-C-2.0|LFP cell at 100 °C with differing rates.

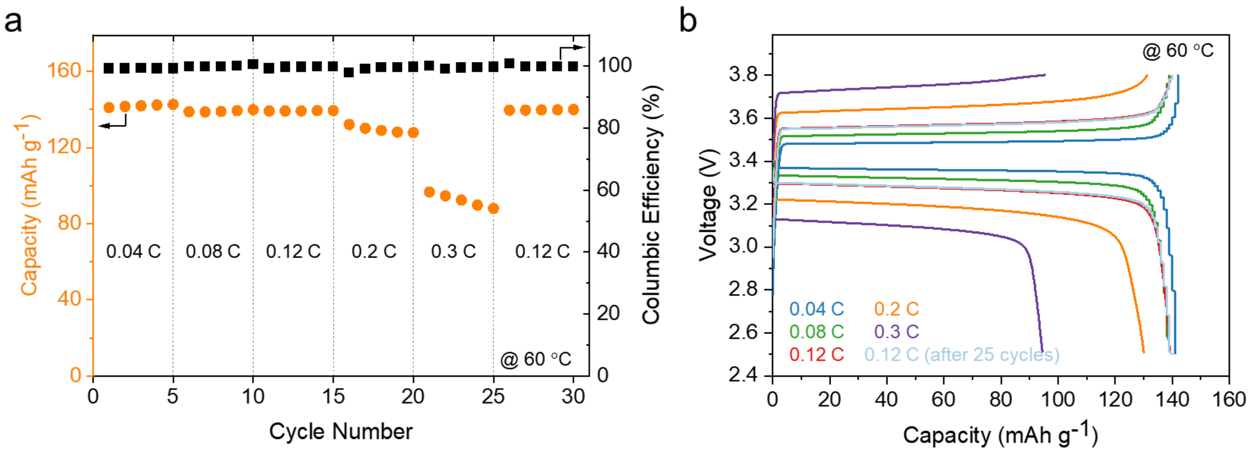

Supplementary Figure 18 | a, Capacity performance and **b**, corresponding charging and discharging profiles of Li|ANP-C-2.0|LFP cell at 60 °C with differing rates.

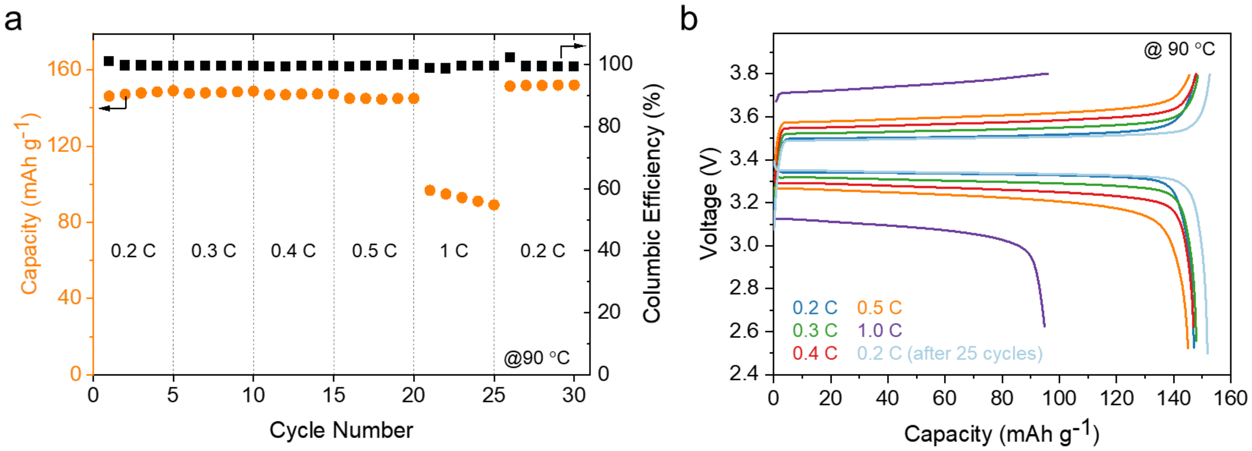

Supplementary Figure 19 | a, Capacity performance and **b**, corresponding charging and discharging profiles of Li|ANP-C-2.0|LFP cell at 90 °C with differing rates.

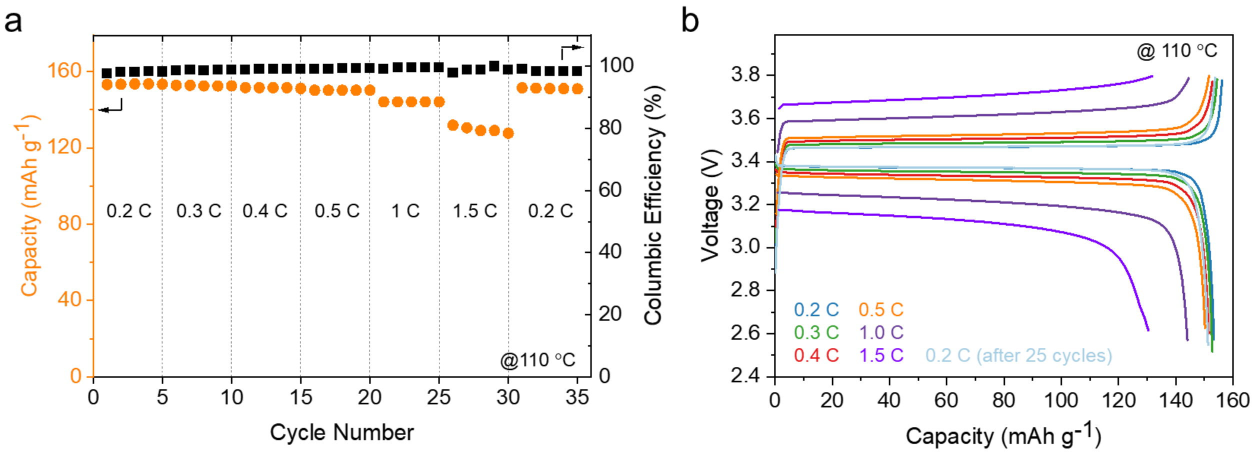

Supplementary Figure 20 | a, Capacity performance and **b**, corresponding charging and discharging profiles of Li|ANP-C-2.0|LFP cell at 110 °C with differing rates.

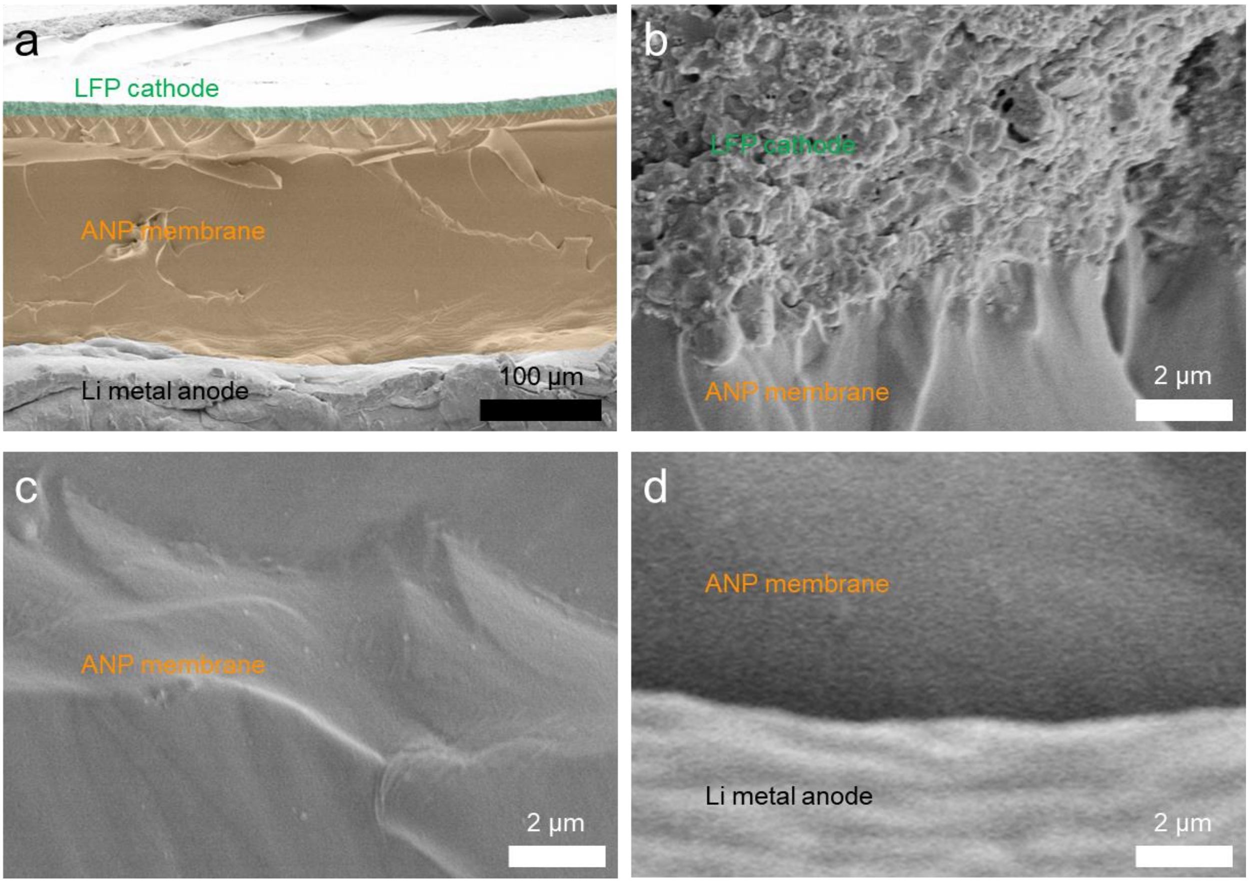

Supplementary Figure 21 | Cross-sectional SEM images of Li|ANP-C-2.0k|LFP cell after battery cycling.

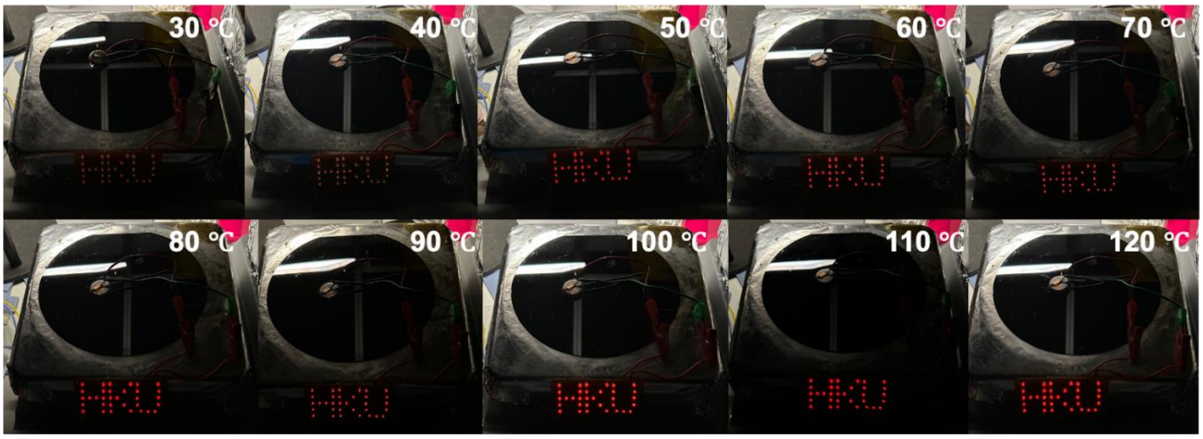

Supplementary Figure 22 | Thermal abuse test of 525 cycled-coin cell upon continuous heating.

Material synthesis

General information Syntheses of borate monomer were conducted under a dry Ar atmosphere via standard Schlenk techniques. Anhydrous diethyl ether, tetrahydrofuran (THF), 1,4-dioxane and SH-PEG-SH (Mn=~1k) were purchased from Sigma-Aldrich. Organic solvents were dried with molecular sieves (4 Å) to further remove water trace. 5-Hexen-1-ol was purchased from TCI and then store with 4 Å molecular sieves at least 48 h before use. SH-PEG-SH (Mn=~1.5k, 2k, 3.4k, 5k) were bought from Aladdin. All other chemicals were purchased from commercial vendors and used as received without further purification. The ^{1}H, ^{13}C, and ^{19}F nuclear magnetic resonance (NMR) spectra were recorded by Bruker AV 400 and 600 MHz spectrometer at room temperature. Samples were dissolved in MeCN-d_3, $CDCl_3$ or DMSO-d_6. FT-IR spectra were collected using PerkinElmer Spectrum two. Thermogravimetric analysis (TGA) was carried out at a heating rate of 10 K min^{-1} in a nitrogen flow (20 mL min^{-1}). Differential scanning calorimetry (DSC) was measured by from 4 to 250 °C at a heating rate of 5 °C min^{-1} under nitrogen atmosphere. X-ray diffraction (XRD) spectra was collected by Rigaku. Strain stress was conducted by Rheometer AERS G2 tensile tester.

Synthesis of 2,3,5,6-tetrafluorobenzyl chloride In an oven-dried Schlenk flask, 2,3,5,6-tetrafluorobenzyl alcohol (10.0 g), tetrabutylammonium chloride (7.8 g), and thionyl chloride (23.0 mL) were added separately. The solution was mixed at 85 °C for 2 hours. After cooling down to room temperature, the flask was placed in a 0 °C ice bath. 40 mL of deionized water was added to the flask to quench the reaction. Then, concentrated aqueous Na_2CO_3 (40 mL) was slowly added to the solution, followed by solid Na_2CO_3 to adjust the pH to 6. The solution was extracted with 40 mL of diethyl ether four times. The organic layer was collected, washed with 40 mL of brine, and dried with $MgSO_4$. After removing the diethyl ether using rotary evaporation under reduced pressure, a yellow oil was obtained. Finally, the

oil was harvested in a flask placed in liquid N2 through vacuum distillation, resulting in a transparent oil.Yield: 8.9 g (86%).

Synthesis of lithium tetrakis(4-(chloromethyl)-2,3,5,6-tetrafluorophenyl)borate (Monomer) In a dry Schlenk flask, tetrafluorobenzyl chloride (2.01 g, 10.1 mmol) was added using a syringe. The flask was then filled with 100 mL of anhydrous diethyl ether through a cannula. The flask was placed in a dry ice bath at -78 °C. Slowly, 4.93 mL (9.8 mmol) of 2 M n-butyllithium in hexanes was added to the solution using a syringe. After one hour, 1.0 M boron trichloride in heptanes (2.24 mL) was added dropwise using a syringe. The solution was stirred at -78 °C for 2 hours and then allowed to warm up to room temperature as the dry ice evaporated. After 18 hours, the reaction was quenched by adding 30 mL of a 0.1 M aqueous LiCl. The organic layer was collected, washed twice with 30 mL of 0.1 M LiCl aqueous solution, and then dried with $MgSO_4$. The resulting pale yellow oil was concentrated using a rotary evaporator. The oil was transferred to a vial and dissolved in 1 mL of dichloromethane. To purify the solution, it was precipitated in hexane three times. Finally, the trace solvent was removed under vacuum, resulting in a white-yellow solid powder. Yield: 1.3 g (65 %).

Synthesis of lithium tetrakis(4-(hexenyloxy)methyl)-2,3,5,6-tetrafluorophenyl)borate Lithium tetrakis(4-(chloromethyl)-2,3,5,6-tetrafluorophenyl)borate (100 mg) was transferred into a vial and heated at 60 °C under vacuum overnight to fully remove water before use. 5-Hexen-1-ol (240 µL) was added into a 50 mL oven-dried Schlenk flask. 1.4 mL THF was transferred to dissolve the linker via syringe. Then, 1.5 mL of 2 M n-butyllithium in hexanes was slowly added into the solution at room temperature with stirring. After 1 h, the THF was removed by vacuum and replaced by anhydrous 1,4-dioxane (2 mL). Then, borate monomers were dissolved in 2 mL of anhydrous 1,4-dioxane and transferred into the flask via syringe. And the vial was rinsed with additional anhydrous 1,4-dioxane (1 mL) and the solution was then added into the flask with stirring. The flask was placed into an oil bath at 80 °C while

stirring under Ar. After 48 h, the solution was then filtered, and yellow solution was transferred into a vial. After evaporating solvent, the remained yellow product was dissolved in 1 mL of dichloromethane. The solution was purified by precipitation in hexane three times. yellow solid product was obtained after the removal of trace solvent under vacuum. Finally, the product was dried at 60 °C under vacuum overnight. Yield: 42 mg (42 %).

ANP-C-1.0K SH-$(OCH_2CH_2)_n$-CH_2CH_2-SH (Mn = 1k, n=20–21) was used as the linker in precursor solution. The product is a yellow membrane. Anal. Calcd. (%) for $C_{138}H_{229}BF_{16}LiO_{24}S_4$: C, 54.14; H, 7.46. Found: C, 53.90; H,8.23%.

ANP-C-1.5K SH-$(OCH_2CH_2)_n$-CH_2CH_2-SH (Mn = 1.5k, n=31–32) was used as the linker in precursor solution. The product is a yellow membrane. Anal. Calcd. (%) for $C_{184}H_{320}BF_{16}LiO_{36}S_4$: C, 54.23; H, 7.86. Found: C, 53.61; H,8.40%.

ANP-C-2.0K SH-$(OCH_2CH_2)_n$-CH_2CH_2-SH (Mn = 2k, n=43–44) was used as the linker in precursor solution. The product is a yellow membrane. Anal. Calcd. (%) for $C_{229}H_{411}BF_{16}LiO_{47}S_4$: C, 54.30; H, 8.10. Found: C, 54.12; H,9.10%.

ANP-C-3.4K SH-$(OCH_2CH_2)_n$-CH_2CH_2-SH (Mn = 3.4k, n=75–76) was used as the linker in precursor solution. The product is a yellow membrane. Anal. Calcd. (%) for $C_{357}H_{665}BF_{16}LiO_{79}S_4$: C, 54.38; H, 8.45. Found: C, 55.20; H,9.23%.

ANP-C-5.0K SH-$(OCH_2CH_2)_n$-CH_2CH_2-SH (Mn = 5k, n=111–112) was used as the linker in precursor solution. The product is a slight yellow membrane. Anal. Calcd. (%) for $C_{502}H_{956}BF_{16}LiO_{115}S_4$: C, 54.43; H, 8.63. Found: C, 53.96; H,9.09%.

Powder ANP PEG (Mn = 1k) was used for synthesis of powder ANP according to our previous work.[S1]

Electrochemical Characterizations

Sample preparations All electrolyte membrane were totally dried at 120 °C under vacuum at least 24 h before being transferred to the glove box filled with Ar. Before electrochemical or battery measurements, all membrane should be stored at least 24 h in the glove box.

Ionic Conductivity Measurement Measuring the ionic conductivity of lithium-ion polymer electrolytes commonly involves using the AC impedance method. This method entails applying a small sine wave of specific amplitude to the system and obtaining the impedance spectrum by varying the frequency. Typically, the Amiral Squidstat Plus is used to investigate the ionic conductivity within an argon-filled glove box. The sample is enclosed within stainless-steel electrodes placed in a Swagelok cell. By applying a 100-mV ac to the Swagelok cell within a frequency range of 1 MHz to 1 Hz, the ionic conductivity of the sample can be calculated using the provided equation.

$$\sigma = \frac{l}{RS}$$

where l is the sample thickness, S indicates the area of sample, and R refers to the bulk resistance. The Swagelok cell, containing the electrolyte, was assembled and then placed in the Belektronig BTC-LAB-A20 temperature controller for temperature-dependent measurements. Impedance spectra were collected at 28 °C, 38 °C, 48 °C, 58 °C, 68 °C, 78 °C, and 88 ° C, with three measurements taken at each temperature. The variable-temperature ionic conductivities were determined using the Arrhenius and Nernst-Einstein equations.

$$\sigma T = \sigma_0 exp\left(\frac{-E_a}{RT}\right)$$

where R is gas constant. E_a and T mean the activation energy and absolute temperature, respectively.

Lithium Transference Number (LTN) The ability of immobilizing anions is shown by LTN, which is an important factor in single-ion polymer electrolytes. It is ideal for the lithium transference number to be close to one, and this can be achieved through structural and electrochemical methods. Determination of LTN in polymer electrolytes is done using the steady-state current method. Ar-filled glove box was used to build symmetric Li | electrolyte| Li Swagelok cells. After allowing the Swagelok cell to reach equilibrium overnight, the impedance spectrum was recorded at 100 mV ac. Subsequently, a dc voltage of 100 mV was

applied and the current response was measured for 2 h. Following the DC polarization, the resistances of the electrolyte and the interface were measured using AC impedance. The LTN value was obtained using the given equation.

$$t_{+} = \frac{I^{s}R_{b}^{s}(\Delta V - I^{0}R_{ct}^{0})}{I^{0}R_{b}^{0}(\Delta V - I^{s}R_{ct}^{s})}$$

in which ΔV means the DC voltage; $R_b^0$and R_b^s are the bulk resistance before and after applying the voltage; $I^0$and I^s indicate the initial current and steady-state current; R_{ct}^0 is the charge transfer resistance before the voltage step while R_{ct}^s is the resistance after polarization.

Cyclic voltammetry To determine the electrochemical working window of polymer electrolytes, a three-electrode Swagelok cell was utilized. The stainless steel served as the working electrode, while the counter electrode and reference electrode consisted of lithium chips. The investigation of the electrochemical stability window involved performing voltage sweeps from -0.5 to 5.5 V at a rate of 0.3 mV s^{-1}.

Galvanostatic polarization The interfacial stability of electrolytes was studied by using a Li | electrolyte membrane | Li Swagelok cell charged/discharged at selected current densities (0.1 and 0.2 mA cm^{-2}) for 3 h per interval at 60 and 100 °C.

Molecular dynamics (MD) simulation

MD simulations were carried out using Materials Studio 2020, and the Universal force field was used to simulate interatomic interactions because of its flexibility to a broad spectrum of systems such as organic molecules and metal complexes [S2]. The whole borate group and Li^+ cation were set as one negative charge and one positive charge, respectively. The partial charges of the borate nodes and polyether linkers were calculated by RESP method [S3] implanted in Multiwfn software [S4]. The wave function and the optimized structure for the RESP calculation were generated in wB97M-V/def2-TVZP level by the ORCA package [S5]. After setting the charges, the simulations were carried out in the isothermal-isobaric condition (with a constant numbers of atoms, constant pressure and constant temperature, NPT). Unless

otherwise specified, a time step of 1fs was used for all simulations. The Andersen thermostat [S6] was utilized for temperature control, while the Berendsen barostat [S7] was used for pressure control. Newton's equation was integrated using the Verlet algorithm. Van der Waals interactions were computed using atom-based summation with a cut-off distance of 9.5 Å, and electrostatic interactions were computed using the Ewald summation.

The initial simulation structures were built by Materials visualizer which is embedded inside the software. Each simulation contained 8 Li^+ atoms and 8 anionic networks. The systems were heated to 343 K for at least 2 ns to simulate the experimental preparation temperature. After the structure shrinks to the experimental density, to achieve ion equilibrium, annealing procedures were employed. The systems were carried out from 298 to 600 K 5 times with each interval of 50 K lasting 50 ps. Simulations of Li transport under applied voltage used potentials of strength between 4 and 8 V nm^{-1} along the z axis. Snapshots of the trajectory were recorded every 1 fs.

In order to study the effective of the Li^+ transportation, the mean square displacement (MSD) method was carried out to investigate the movements of atoms and molecular segments of the systems [S8]. The MSD can be obtained from the position change of particles in unit time in a molecular dynamics (MD) simulation by following equation:

$$MSD = \frac{1}{\tau - \Delta t}\int_0^{\tau-\Delta t} [r(t-\Delta t) - r(t)]^2 dt$$

where τ represents the total production time and r(t) is the position at time t.

The radial distribution function g(r) was calculated as:

$$g(r) = \frac{dn_r}{\rho 4\pi r^2 dr}$$

where n_r is the atom number in the spherical shell, ρ is the number density of the whole system and r is the distance.

The Binding energy between the Li+ atoms and the network systems was calculated as:

$E_{interaction} = E_{total} - (E_{Li} + E_{network})$

where the E_{total}, E_{Li}, and $E_{network}$ are the whole systems energy, the Li^+ energy, and the network energy in the periodic structure, respectively.

Supplementary References

[S1] J. Gao, J. Zhou, C. Wang, X. Ma, K. Jiang, E. Kim, C. Li, H. Liu, L. Xu, H. C. Shum, S.-P. Feng, D.-M. Shin, Chem. Eng. J. 2022, **450**, 138407.

[S2] A. K. Rappe, C. J. Casewit, K. S. Colwell, W. A. Goddard and W. M. Skiff, *J. Am. Chem. Soc.*, 1992, **114**, 10024-10035.

[S3] Wang, J., Cieplak, P. & Kollman, P. A. *J. Comput. Chem.* **21**, 1049–1074 (2000).

[S4] Lu, T. & Chen, F. *J. Comput. Chem.* **33**, 580–592 (2012).

[S5] Neese, F., Wennmohs, F., Becker, U. & Riplinger, C. *J. Chem. Phys.* **152**, 224108 (2020).

[S6] G. J. Martyna, D. J. Tobias and M. L. Klein, *J. Chem. Phys.*, 1994, **101**, 4177-4189.

[S7] H. J. C. Berendsen, J. P. M. Postma, W. F. van Gunsteren, A. DiNola and J. R. Haak, *J. Chem. Phys.*, 1984, **81**, 3684-3690.

[S8] Q. Xue, C. Lv, M. Shan, H. Zhang, C. Ling, X. Zhou and Z. Jiao, *Comput. Mater. Sci*, 2013, **71**, 66-71.

Supplementary Movie

Supplementary Movie 1 | Snapshots of Li^+ cation conduction throughout anionic network polymer.

Supplementary Movie 2 | Flame test of propylene carbonate and ANP-C-2.0K membrane.
